# Distribution and diversity of ribosome binding sites in prokaryotic genomes

**DOI:** 10.1186/s12864-015-1808-6

**Published:** 2015-08-14

**Authors:** Damilola Omotajo, Travis Tate, Hyuk Cho, Madhusudan Choudhary

**Affiliations:** Department of Biological Sciences, Sam Houston State University, Huntsville, TX 77341 USA; Department of Computer Science, Sam Houston State University, Huntsville, TX 77341 USA

**Keywords:** Shine-Dalgarno sequence, Ribosome binding site, Translation initiation

## Abstract

**Background:**

Prokaryotic translation initiation involves the proper docking, anchoring, and accommodation of mRNA to the 30S ribosomal subunit. Three initiation factors (IF1, IF2, and IF3) and some ribosomal proteins mediate the assembly and activation of the translation initiation complex. Although the interaction between Shine-Dalgarno (SD) sequence and its complementary sequence in the 16S rRNA is important in initiation, some genes lacking an SD ribosome binding site (RBS) are still well expressed. The objective of this study is to examine the pattern of distribution and diversity of RBS in fully sequenced bacterial genomes. The following three hypotheses were tested: SD motifs are prevalent in bacterial genomes; all previously identified SD motifs are uniformly distributed across prokaryotes; and genes with specific cluster of orthologous gene (COG) functions differ in their use of SD motifs.

**Results:**

Data for 2,458 bacterial genomes, previously generated by Prodigal (PROkaryotic DYnamic programming Gene-finding ALgorithm) and currently available at the National Center for Biotechnology Information (NCBI), were analyzed. Of the total genes examined, ~77.0 % use an SD RBS, while ~23.0 % have no RBS. Majority of the genes with the most common SD motifs are distributed in a manner that is representative of their abundance for each COG functional category, while motifs 13 (5′-GGA-3′/5′-GAG-3′/5′-AGG-3′) and 27 (5′-AGGAGG-3′) appear to be predominantly used by genes for information storage and processing, and translation and ribosome biogenesis, respectively.

**Conclusion:**

These findings suggest that an SD sequence is not obligatory for translation initiation; instead, other signals, such as the RBS spacer, may have an overarching influence on translation of mRNAs. Subsequent analyses of the 5′ secondary structure of these mRNAs may provide further insight into the translation initiation mechanism.

## Background

A myriad of genetic and biochemical analyses had been carried out to elucidate the regulation of protein biosynthesis in prokaryotes, with most of these studies being centered on translation initiation – the limiting step of translation [1]. The translation initiation process involves the docking, anchoring, and accommodation of mRNA to the mRNA channel of the small 30S ribosomal subunit, and the recruitment of fMet-tRNA to the P-site of the activated mRNA-30S initiation complex [2]. This orderly assembly is facilitated by initiation factors (IF1, IF2, and IF3), which prevent untimely re-association of the ribosomal subunits and promote activation of the initiation complex. Ribosomes consist of two-thirds rRNAs and one-third ribosomal proteins [3]. Some ribosomal proteins in the 30S subunit serve to organize and stabilize the tertiary structure of the 16S rRNA, while other proteins (S2, S7, S8, S11, and S21) act as helicase to unfold mRNA during accommodation to the ribosome. The factors that affect the formation of the 30S initiation complex include the ribosome binding site (RBS) spacer (the distance between the start codon and the RBS), the non-random distribution of nucleotides upstream of the start codon, and the secondary structure of mRNA. The secondary structure of mRNA causes a delay in mRNA accommodation, which could promote the binding of translation repressor proteins to inhibit translation [2, 4].

There are several potential initiation sites in an mRNA competing for a limited number of 30S subunits. It has been proposed that there is a purine-rich sequence similar to 5′-GGAGG-3′, the Shine Dalgarno (SD) sequence, located ~5–10 nucleotides upstream of the start codon in the mRNA [5]. The SD sequence is shown to function as a region with high affinity for the 30S subunit binding, and aid in the selection of the correct translational reading frame [6–8]. Also, there is a highly conserved sequence located near the 3′ end of the 16S rRNA that complementarily binds to the SD sequence to facilitate mRNA anchoring and adaptation to the 30S ribosome [9]. Furthermore, the SD-16S rRNA interaction promotes unfolding of the secondary structure in the leader sequence to make the start codon more accessible. Mutation of the SD motif and/or its complementary sequence in the 16S rRNA has been shown to severely reduce the level of protein synthesis in *Escherichia coli* [10]. While the SD motif was thought to be universal in prokaryotes, alternate forms of RBS (non-SD motifs) have also been discovered in many species of prokaryotes. For instance, archaeal genomes have shown a strong conservation of a 5′-GGTG-3′ atypical RBS within 15 nucleotides upstream of the start site, as well as a loss of 3′ terminal nucleotides of the 16S rRNA [11]. Furthermore, some genes, like *rps*A in *Escherichia coli*, have no consensus RBS (neither SD nor non-SD) in their leader sequences; however, their mRNAs are translated as efficiently as those that possess RBS [12]. This may indicate the presence of some undiscovered factor(s) for the translation of mRNAs with no consensus RBS. In several cases (like in *cyanobacteria*), AT-rich motifs, instead of an SD sequence, are found upstream. Although it is unknown whether these are genuine RBS motifs, a previous study has suggested that the ribosomal protein S1 binds to these AT-rich regions and unfolds the leader sequence of mRNA to make the start codon readily accessible [13]. Also, some archaeal and eubacterial species contain leaderless transcripts that may have evolved to have more accessible start codons [11]. In contrast to prokaryotic mRNAs, all eukaryotic mRNAs are leaderless and utilize a different translation initiation mechanism, where a translation initiation signal, the Kozak sequence [14], is embedded around the start codon.

This study is aimed at assessing the distribution and diversity of RBSs among different groups of prokaryotes to understand the implication of an RBS in the translation initiation mechanism. The following three hypotheses were tested. First, SD motifs are prevalent in bacterial genomes. Second, all previously identified SD motifs are uniformly distributed across prokaryotes. Third, genes with specific cluster of orthologous gene (COG) functions differ in their use of SD motifs.

A total of 2,458 fully annotated genomes, representing eubacterial and archaebacterial groups, were examined in this study. The Protein Table file (.ptt) and the corresponding gene prediction file (.Prodigal-2.50) by Prodigal (PROkaryotic DYnamic programming Gene-finding ALgorithm) [15] for each replicon were downloaded from NCBI FTP directory (ftp://ftp.ncbi.nlm.nih.gov/genomes/Bacteria/). For each replicon, genes commonly present in both the Protein Table files and the Prodigal files were targeted to minimize false positive gene selection. For each selected gene, the following information was collected and organized for the study: taxonomy, replicon (chromosome or plasmid), RBS type (SD motif or no RBS), RBS spacer, and COG functions.

## Results and discussion

### Prevalence of RBS types among prokaryotic genes

Since most prokaryotic genomes are organized in operons, the first gene of the operon will contain a leader sequence while the subsequent genes within that operon will be leaderless. Gene operons of the bacterial genomes have not been completely annotated so that we are able to differentiate the genes with and without the leader sequence. Thus, in this study, the following two classes of genes are considered: genes with a leader sequence containing an SD motif and genes with no RBS motifs.

**Fig 1:**
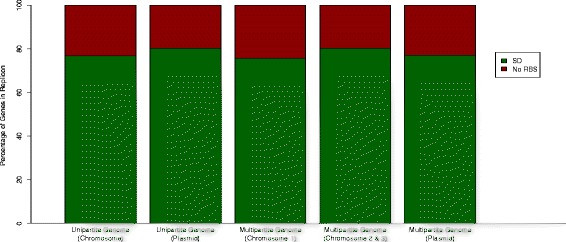
Relative Abundance of Genes with SD or No RBS in Bacterial Genomes. Data is organized into replicon groups (Chromosome I, Chromosome II, Chromosome III, and Plasmid) and then separated based on genome complexity (unipartite genome and multipartite genome)

**Fig 2:**
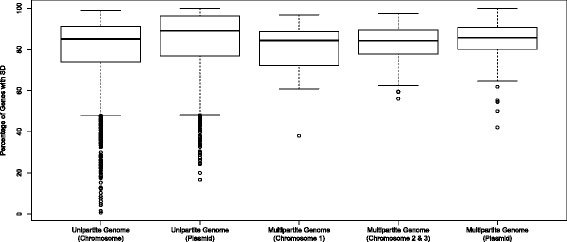
Distribution of genes with SD in Bacterial Genomes. The data is organized in the same fashion as Fig. 1; however, only SD motifs are considered. Circles denote outliers and the dark bolded line within each box (inter-quartile range) denotes the median

Figure 1 displays the distribution of genes with an SD motif and those with no RBS among unipartite and multipartite genomes. On average, genes with an SD motif and no RBS represented ~77 % and ~23 %, respectively. A previous study on ~800 prokaryotic genomes reported that ~88 % genes have SD motifs [15]. However, the lower percentage of SD RBS (77 %) identified in this study could be attributed to a large sample (2,458 genomes) that represents diverse prokaryotic groups, analyses of more extreme genomes, or changes in Prodigal gene finder over time. It has been reported that the majority of the genes from archaebacterial species encode leaderless mRNAs, lacking RBS motifs [11]; however, our result showed that 34 (out of 2,307) eubacterial genomes and 29 (out of 151) archaeal genomes contain genes that lack an RBS. Therefore, genes with no RBS are present in both eubacteria and archaebacteria. The distribution of SD usage is shown in Fig. 2, where species are separated based on unipartite and multipartite genome organization. The data consists of 2,343 (~95 %) unipartite and 115 (~5 %) multipartite genomes. Organisms with unipartite genomes (with a single chromosome) have fewer genes with an SD RBS in comparison to organisms with multipartite genomes (with more than one chromosome) based on the wider interquartile range and higher percentage of outliers in unipartite genome (p < 0.001, Kruskal Wallis test).

A significant difference in medians (*p* < 0.009) was seen among the multipartite replicon groups (Fig. 2); however, no significant difference in variance (*p* = 0.192, Levene’s test) was observed across the three groups.

Post-hoc analyses revealed a significant difference between multipartite primary chromosome and secondary chromosomes (*p* = 0.009, Mann Whitney test) as well as a significant difference between multipartite primary chromosome and multipartite plasmids (*p* = 0.014). However, no significant difference between multipartite secondary chromosomes and plasmids was observed (*p* = 0.199). This suggests that, within the multipartite classifications, the primary chromosomes may have diverged in the use of SD motifs in comparison to the other two groups, with the secondary chromosomes and plasmids being more similar in the use of an SD RBS.

Out of the 2,458 genomes, 1,444 (~58.7 %) use an SD RBS strongly (containing ≥80 % genes with an SD sequence), 695 (~28.3 %) moderately (containing ~40–79 % genes with an SD sequence), and 75 (~3 %) use an SD RBS minimally (containing ~18–39 % genes with an SD sequence). This distribution, however, is more representative of unipartite genomes. Over 40 % of genes in multipartite genomes have an SD sequence. The remaining 10 % (244 genomes) of prokaryotes (including some *bacteroidetes, cyanobacteria, crenarchaea*, and *nanoarchaea*) do not use a consensus SD sequence.

It is worth noting that the multipartite archaebacteria, *Haloarcula hispanica, Haloarcula marismortui and Halorubrum lacusprofundi*, do not use any form of known RBS. A possible explanation for this result is that most archaebacteria tend to lack a 5′-untranslated region (UTR); thus, they do not have a probable RBS [13, 16]. Our observation of unipartite archaebacteria (148 genomes), on the other hand, revealed that 3 (~2 %) contain a low percentage (~18–38 %) of genes with an SD motif, 77 (~52 %) contain a medium to high percentage (~53–85 %) of genes with an SD motif, and 68 (~46 %) contain genes with no RBS. This could suggest that different evolutionary pressures may operate in archaebacteria that use an SD RBS or no RBS (use of non-SD motifs or lack of a 5′-UTR like eukaryotes). Separating the archaeal group from prokaryotes did not affect the wide distribution of eubacterial organisms that utilize an SD RBS or the number of outliers in the unipartite replicons. This, again, implicates a lower SD RBS conservation among unipartite genomes.

### Conservation of SD RBS within replicons

Further analysis was performed to understand the conservation of SD RBS among genes within different chromosomal and plasmid replicons. About 68 % (1,282 out of 1,889) of plasmids and ~62 % (1,446 out of 2,343) of chromosomes use SD motifs strongly (≥80 % genes with SD RBS) for unipartite genomes. This distribution is similar in multipartite genomes since ~67 % (63 out of 94) of plasmids and ~56 % (138 out of 246) of chromosomes use SD motifs strongly. Upon comparing primary chromosomes (with more essential genes) and secondary chromosomes of multipartite genomes, it was found that secondary chromosomes have ~60 % (78 out of 131) of genes with an SD motif while primary chromosomes contain ~52 % (60 out of 115) of these genes. This, alongside the results from the statistical tests conducted previously, suggests that in bacterial genomes, SD RBS is associated with fewer essential genes even though a majority of genes possess an SD motif. This inference, however, still requires further analysis and experimental validation.

### Abundance of putative non-SD RBS in prokaryotes

Generally, SD-16S rRNA interactions are known, not only to impact ribosome stability and initiation site selection, but also to help reduce secondary structure formation around the start codon to promote translation efficiency. In fact, studies have shown that SD sequence-containing mRNAs have lower folding energies [17, 18]. The presence of non-SD RBS has not been fully explored with respect to translation efficiency. Previous studies have shown that the ribosomal protein S1 acts independent of an SD sequence, and binds to non-SD motifs to mediate translation initiation in *E. coli* [13, 18]; however, the S1 protein does not appear to be essential in many other prokaryotes [19].

**Fig 3:**
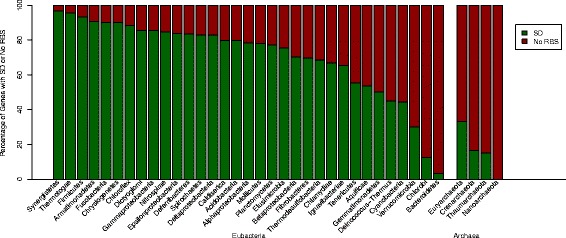
Comparison of Distribution of Genes with SD or No RBS among Different Bacterial Groups. The data is organized into various prokaryotic groups and separated into two larger groups, Eubacteria and Archaea

Figure 3 shows that the percentage of genes with no RBS (some of which might contain atypical binding sites) is especially high in prokaryotes that are metabolically diverse like *Bacteroidetes* –or are well adapted extremophiles like *Chlorobi*, *Deinococcus-Thermus*, C*yanobacteria*, and Archaea. Particularly, *Bacteroidetes* have highly plastic genomes that are able to undergo frequent genetic rearrangement to adapt to several ecological niches ranging from soil to large intestine in humans [20] – According to Kozak [14], the non-SD RBS forms weak secondary structures, promoting easier access of the ribosome to the start codon. This could explain why there is no observable loss in translation initiation efficiency of mRNAs with no RBS. However, it has not been confirmed whether these motifs are contained exclusively in the mRNA leader sequences [13]. It is quite possible that the presence of a non-SD motif downstream may equally influence the melting of the secondary structure of mRNAs.

### Diversity of organisms with genes that have no RBS

Many genes in *Cyanobacteria* and *Bacteroidetes* have no consensus RBS in their leader sequences. It is also believed that there are a large proportion of leaderless mRNAs, with no RBS [21], frequently occurring in single genes and proximal operon genes of prokaryotes [13]. While translation mechanisms for leaderless mRNAs are poorly understood, some reports have postulated that leaderless mRNAs undergo translation initiation in the presence of 70S monosomes (preassembled 30S-50S ribosomes) and IF2 in thermophiles [22–24]. It has been shown that protein synthesis could be hindered by steric interference of secondary structures present in the 5′-UTR of the mRNA [14]; thus, it is possible that these leaderless mRNAs could be more efficient in translation than the canonical mRNAs.

### Genomic composition does not affect RBS type

Previous finding revealed that the presence of an SD sequence is positively correlated with the GC content of an organism [18]. However, some microorganisms, such as *Firmicutes and Fusobacteria*, have low % GC content in their genomes [25], but contain a high percentage of genes with an SD RBS. On the contrary, organisms like *Bacteroidetes* and *Chlorobi* with relatively higher % GC genome composition have very low percentage of genes with an SD RBS motif (Fig. 3).

### Distribution of different SD motifs across prokaryotes

**Fig 4:**
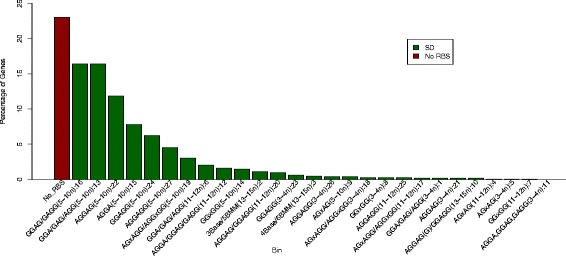
Bacterial Usage of SD Motifs. The bins are arranged in decreasing order of percentage of genes with the corresponding motif(s). Each bin except “No_RBS” is labeled with the SD sequence(s), the spacer from the start codon, and the corresponding bin number assigned by Prodigal [15]

The SD motif types, as identified by Prodigal, were analyzed for their distribution over all prokaryotes. The relative frequency of each of the 27 SD motifs in prokaryotes is displayed in Fig. 4, where the top seven mostly utilized SD motifs, corresponding to bins 16, 13, 22, 15, 24, 27, and 19, have a 5-10n RBS spacer. Regardless of the bacterial group and the genome complexity (unipartite or multipartite genome), these seven motifs are mostly used across species. Specifically, about 86 % of genes with an SD sequence use these seven motifs. Studies have identified the −4 to +30 region of the mRNA as a region critical for ribosome binding, wherein stability has to be minimized for efficient translation initiation [11, 26–28]. Although the exact distance of the RBS from the start codon is not fully ascertained in this study; it is likely that a 5-10n RBS spacer is important, as an RBS further away from the start codon could be less efficient in melting secondary structure in the critical region. Nonetheless, it would be helpful to perform a follow-up analysis, which does not completely rely on Prodigal’s output available on NCBI, to obtain precise spacer lengths and, possibly, elucidate the implication of the RBS spacer on translation initiation.

### COG function analysis of genes with an SD RBS

**Fig 5:**
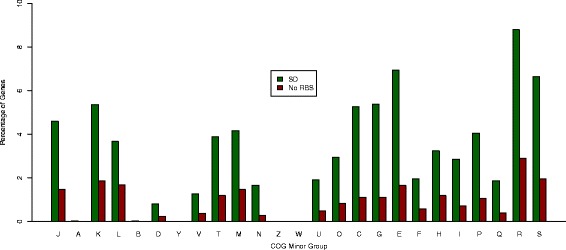
Distribution of Genes with SD or No RBS over COG Minor Groups. A simple script is used to tally up the frequency of 25 COG minor categories for every RBS motif in every replicon. The minor subgroups fall under the major groups: COG 1. Information storage and processing; COG 2. Cellular processing and signaling; COG 3. Metabolism; and COG 4. Poorly characterized functions. These subgroups include: J. Translation, ribosomal structure and biogenesis (COG 1); A. RNA processing and modification (COG 1); K. Transcription (COG 1); L. Replication, recombination and repair (COG 1); B. Chromatin structure and dynamics (COG 1); D. Cell division and chromosome partitioning (COG 2); Y. Nuclear structure (COG 2); V. Defense mechanisms (COG 2); T. Signal transduction mechanisms (COG 2); M. Cell wall/membrane/envelope biogenesis (COG 2); N. Cell motility (COG 2); Z. Cytoskeleton (COG 2); W. Extracellular structures (COG 2); U. Intracellular trafficking, vesicular transport and secretion (COG 2); O. Posttranslational modification, protein turnover, chaperones (COG 2); C. Energy production and conversion (COG 3); G. Carbohydrate transport and metabolism (COG 3); E. Amino acid transport and metabolism (COG 3); F. Nucleotide transport and metabolism (COG 3); H. Coenzyme metabolism (COG 3); I. Lipid metabolism (COG 3); P. Inorganic ion transport and metabolism (COG 3); Q. Secondary metabolites biosynthesis, transport and catabolism (COG 3); R. General function prediction only (COG 4); and S. Function unknown (COG 4) [31]

Although many genes have not been assigned COG functions, many are poorly characterized, and, still, many have been assigned more than one COG function, the COG function data in publicly-available NCBI Protein Table files (.ptt) was used for this analysis. Results indicate that the relative frequency of genes with an SD sequence is significantly higher than that of genes with no RBS (p < 2.2 e^−16^, Chi-squared test) for all minor COG functional groups except the COG subgroup, Y (nuclear structure) (p = 0.5637). In particular, one gene with an SD motif, AGGAG: 5-10n: 22, and two genes with no RBS were predicted to fall under the functional category, Y. Figure 5 shows that there are also very few genes (<0.05 % of genes with an SD motif or no RBS) that are in COG functional groups A, B, Z and W, which correspond to RNA processing and modification, Chromatin structure and dynamics, Cytoskeleton, and Extracellular structures, respectively. This is expected since all features listed are predominantly eukaryotic features.

Furthermore, genes with the seven most common SD motifs were examined to ascertain any COG functional specialization. It was found that genes with these seven SD motifs are not evenly distributed in the five major COG functional categories. On the average, genes representing no function (COG 0) and unknown or poorly characterized functions (COG 4) constitute ~50 % and ~10 %, respectively. Genes involved in information storage and processing (COG 1) and cellular processing and signaling (COG 2) constitute a similar level of ~10 %. Genes involved in metabolism (COG 3) are used at a level of ~20 %.

**Fig 6:**
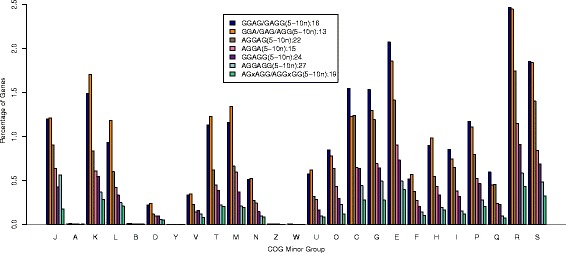
Distribution of Genes with Seven Most Abundant SD Motifs over COG Minor Groups. The COG subgroup classifications are identical to those in Fig. 5. The relative frequency of genes with the seven most abundant motifs within each of the COG minor categories is plotted to see if genes in a COG functional category use a specific SD motif

Specifically, all these seven motifs are used at different levels across the COG subgroups (minor groups) other than subgroups A, B, Y, Z and W (Fig. 6). These motifs are used at a level of ~1–2 % within COG subgroup D (cell cycle control, cell division and chromosome partitioning) within subgroup V (defense mechanisms), subgroup N (cell motility), subgroup U (intracellular transport, secretion and vesicular transport), subgroup F (nucleotide transport/metabolism), and subgroup Q (secondary metabolites biosynthesis, transport/catabolism); ~3–5 % within subgroup J (translation, ribosomal structure/biogenesis), subgroup L (replication, recombination/repair), subgroup T (signal transduction mechanisms), subgroup M (cell wall/membrane/envelope biogenesis), subgroup O (posttranslational modification, protein turnover and chaperones), subgroup H (coenzyme transport/metabolism), subgroup I (lipid transport/metabolism), and subgroup P (inorganic ion transport/metabolism); and ~6–10 % within, subgroup K (transcription), subgroup C (energy production/conversion), subgroup G (carbohydrate transport/metabolism), subgroup E (amino acid transport/metabolism), subgroup R (general function prediction only), and subgroup S (unknown function).

Figure 6 also depicts that the relative abundance of genes with these motifs in each minor COG functional group is positively correlated to their overall abundance from Fig. 4, with a few exceptions. Bin 27 (5′-AGGAGG-3′) is utilized more for the COG subgroup J than bin 24 (5′-GGAGG-3′) (p < 2.2 e ^−16^). In addition, bin 13 (5′-GGA-3′/5′-GAG-3′/5′-AGG-3′) is used more in information storage and processing (specifically COG subgroups K, L, T and M) compared to bin 16 (5′-GGAG/GAGG-3′) (p < 2.2 e ^−16^ for each case).

## Conclusions

We have studied the distribution of ribosome binding sites in 2458 completely sequenced prokaryotic genomes, in order to elucidate the possible impact of the presence and variation of RBS in translation initiation process. Our study with the publicly available NCBI data revealed that ~23 % of bacterial genes lack an RBS. Also, a higher proportion of essential genes in several unipartite and multipartite genomes do not use an SD RBS. This alludes to the obligatory nature of the SD sequence and the possible adaptation of an alternate translation initiation mechanism by prokaryotes. As mRNA stability around the start codon is critical to efficient translation, the RBS spacer might be another factor to consider, other than the presence of an RBS. In addition, majority of genes with a SD sequence have motifs with a 5-10n spacer; therefore, experimental analyses done to see if a change in location of such RBS motifs is detrimental may determine if this RBS spacer is optimal. Furthermore, in most cases, the distribution of SD-containing genes with respect to the COG functional categories is reflective of the relative abundance of these genes overall. However, genes with SD motifs corresponding to bins 13 and 27 appear to be mostly used in major COG group 1 (information storage and processing) and minor COG group J (translation, ribosomal structure/biogenesis) respectively. This indicates that some genes with specific COG functions may differ in their use of an SD RBS.

## Methods

### Gene Prediction Data acquisition from NCBI

NCBI performs automated gene prediction programs such as GeneMark.hmm [29], Glimmer [30], and Prodigal [15] and makes gene prediction results of completely sequenced genomes available in the FTP directory (ftp://ftp.ncbi.nlm.nih.gov/genomes/Bacteria/). Both the gene prediction files (.Prodigal-2.50) and NCBI Protein Table files (.ptt) for 2,754 Prokaryotic genomes were downloaded from the NCBI FTP directory.

Prodigal was designed to achieve the three specific goals – improvement of gene structure prediction, improvement of translation initiation site recognition, and reduction of false positives of gene prediction. Prodigal has been reportedly the most robust gene finder for diverse genomes; thus, the genes predicted by Prodigal were used in this study. Accordingly, our study with the Prodigal prediction genes would be expected to be stringent and robust.

Prodigal scans the entire genome, examining any frame plot bias for Gs and Cs, and builds initial gene models. Hexamer statistics are gathered for each gene model, and a coding score is calculated based on these statistics. Prodigal initially assumes that all protein coding genes have a ribosome binding site to reduce the chance of reporting false negatives, and scans 2–15n regions upstream of the high scoring start codons (ATG, GTG and TTG) to assign an RBS score. Coding peaks representing highest-scoring translation initiation sites are also recorded for each open reading frame (ORF). Then, bin numbers are generated for each initiation site based on RBS score and RBS spacer. Prodigal uses bins 0–27, with 0 indicating the absence of SD motif, the lowest score, and 27 being the highest scoring motif. A more rigorous scanning is done on outer regions (1–2n and 15–45n upstream) for all genes and an upstream score is reported. Genes with AT-rich sequences were found in several organisms and classified in our study as a part of genes with no RBS.

### Data Organization based on Motif, Species Classification, and COG Function

Each Prodigal file contains a list of useful information of each predicted gene for our study, which includes gene location in the considered replicon, start codon, RBS motif, and RBS spacer. Each NCBI Protein Table file contains the profile of each CDS, which includes location, strand, length, PID, gene, synonym, code, COG, and product. Gene location common in both files was used as a key to join these files for each replicon. This joining process can be considered as the intersection operation between the two files to further reduce false positive gene predictions. The resulting files were manually screened so as to remove any discrepancies as follows: a single strain was selected for strains with multiple accession numbers; phages were removed; organisms that did not have fully annotated genomes were also removed; and plasmid sequences not associated with any native organisms were removed. This prescreening step resulted in 2458 genomes (originally 2754), of which 2343 were unipartite (2196 eubacteria and 147 archaebacteria) and 115 were multipartite (112 eubacteria and 3 archaebacteria).

The refined data was then employed to test specific hypotheses. Furthermore, the frequencies of genes with an SD RBS, or lacking RBS motifs, and the corresponding COG functions of these genes were collected. The COG functions of the frequently occurring SD motifs were compared.

### Statistical analyses

A Kruskal-Wallis rank sum test with adjustments for tied ranks, was done to evaluate statistically significant differences in distribution of SD motifs. A nonparametric version of the Levene’s test was also conducted to verify the assumption of homogeneity of variance made for Kruskal-Wallis test. Post-hoc analyses were done using the Mann–Whitney test to evaluate any pairwise differences among the three groups (multipartite primary chromosome, secondary chromosome, and plasmid groups), with Bonferroni correction for Type-1 error. Lastly, a Chi-squared test for equality of proportions (without Yates’ correction for continuity) was performed to determine if the differences in relative abundance of RBS motifs was statistically significant in the COG analyses.

## Abbreviations

SD: Shine-Dalgarno
RBS: Ribosomal Binding Site(s)
non-SD: Alternative motif(s) (putative ribosomal binding sites other than SD)
